# Study protocol: Evaluation of the ‘real-world’ Farmers Have Hearts – Cardiovascular Health Program

**DOI:** 10.1016/j.pmedr.2022.102010

**Published:** 2022-10-17

**Authors:** Diana van Doorn, Noel Richardson, David Meredith, Catherine Blake, John McNamara

**Affiliations:** a National Centre for Men’s Health, South East Technological University, Kilkenny Road, Carlow R93 V960, Ireland; b Rural Economy Development Programme, Teagasc, Ashtown, Dublin 15 D15 DY05, Ireland; c School of Public Health, Physiotherapy & Sports Science, University College Dublin, University College Dublin, Belfield, Dublin 4 D04 V1W8, Ireland; d Kildalton College, Teagasc, Piltown, Co Kilkenny E32 YW08, Ireland

**Keywords:** Gender-specific methodology, ‘hard-to-reach’ population group, Farmers, ‘Real world’ trial, Health behavior change intervention, Study protocol

## Abstract

Premature cardiovascular disease (CVD) mortality among men represents a public health concern worldwide. In Ireland, male farmers are a ‘high-risk’ group for CVD mortality compared to normative values for Irish males. Despite this, they are perceived to be a ‘hard-to-reach’ (HTR) group to engage with health interventions. Primary prevention measures, including health screening and health behavior change interventions, are key strategies in addressing CVD yet often do not reach HTR groups such as male farmers.

The Farmers Have Hearts – Cardiovascular Health Program (FHH-CHP) is a unique large-scale (n = 868) workplace health intervention specifically targeted at Irish male livestock farmers. It included a baseline and Week 52 health check and a health behavior change intervention with three delivery methods: ‘health coach by phone’ and/or mobile (M)-health by text message. The program adopted gender-specific and strengths-based methods to maximize participant engagement. It integrated a multi-actor approach and was trialed in a ‘real world’ practice outside of confined health care settings. Data collection comprised health check results (baseline, Week 52) and self-report measures (baseline, Week 26, Week 52).

The FHH-CHP is the first targeted health intervention adopting gender-specific methods to reach and engage male livestock farmers in their cardiovascular health. Documenting the FHH-CHP study protocol is important therefore and will benefit practitioners attempting to apply gender-specific approaches to engage at-risk and HTR groups of men with targeted health interventions. Meeting men outside of clinical health settings and adopting gender competency standards are needed to address inequities in health outcomes experienced by men.

## Highlights

• Informing gender-specific, community-based men’s health promotion programs.
• Engaging ‘high-risk’ and ‘HTR’ groups’ in cardiovascular disease prevention.
• Cardiovascular health prevention targeted at male farmers.
• Build gender competency in service delivery.
• Addressing inequities in health experienced by sub-population groups of men.

## 1 Introduction

Cardiovascular disease (CVD) represents a public health concern and has strong gendered and social gradient dimensions (World Health Organization European Region Office, 2018). Population screening is the main CVD prevention strategy but is on its own is not effective in reducing risks (Eriksen et al., 2021) and those most at risk, i.e. males who are single, from lower socio-economic status (SES) groups, lower educational attainment and with unhealthy lifestyle behaviors, are least likely to engage in prevention programs (Dryden et al., 2012). Male farmers were found a ‘high-risk group for CVD mortality. This has been found among Australian farm populations compared to non-farm rural populations (Wilder and Brumby, 2012), whilst in Ireland, male farmers were found to have a seven times higher CVD mortality compared to salaried employees (Smyth et al., 2013). In 2020 in Ireland, the agricultural workforce accounted for 278.600 people and included predominantly males: 86.4 % of farm holders and 73 % of agricultural workers were male (Central Statistics Offi Census of Agriculture, 2020). Male farmers are considered a ‘hard-to-reach’ (HTR) group with health interventions because of their rural location, adherence to more traditional masculinity norms, and lower SES (Shaghaghi et al., 2011).

**Fig. 1:**
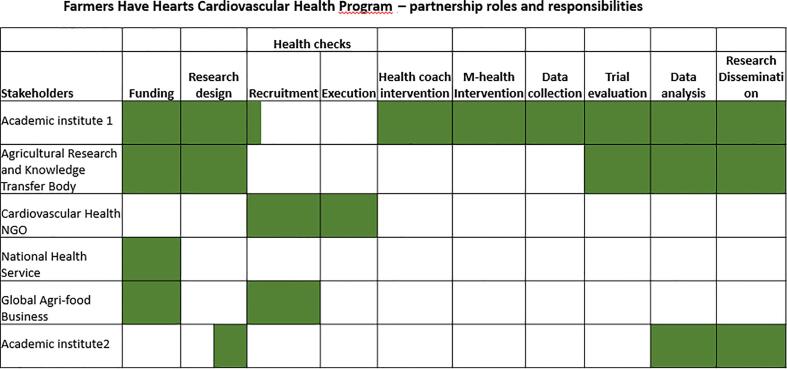
Roles and Responsibilities Farmers Have Hearts Cardiovascular Health Program Partners.

A criticism of many health interventions is their focus on ‘efficacy testing’ in controlled environments and, as a result, their limited application to real-world contexts (Curran et al., 2012). There is a need for evidence-based, ‘real-world’ approaches that successfully engage at-risk populations. Real-world research observes the effects of interventions tested outside of Randomized Control Trials (RTC’s) (Glasgow and Emmons, 2007, Plueschke et al., 2018) and examine program performance when trialed in ever-changing and unpredictable daily situations. Consequently, the learnings are more relatable to health practitioners (Alberts et al., 2014). This paper describes the methodology used in the design, trialing and evaluation of the ‘Farmers Have Hearts Cardiovascular Health Program’ (FHH-CHP). This program adopted a transdisciplinary approach including two academic institutes, a national agricultural research, extension and education organization, a cardiovascular health NGO, the national health service and a global agri-food business. Farmers’ input was generated by semi-structured qualitative interviews (n = 3) after which information redundancy was reached (Vasileiou et al., 2018) as farmers had difficulty envisioning targeted health interventions. It also included reflections of farmers (n = 172) on the design of farmers’ health interventions, collected as part of the FHH pilot evaluation 2013/14 (van Doorn et al., 2019). Farmers (n = 17) also piloted the baseline questionnaire to assess face and content validity. Partners’ roles and responsibilities (Fig. 1) were related to their expertise and were documented in a memorandum of understanding.

The FHH-CHP comprised both health screening and a health behavior change (HBC) intervention. The program adopted a strengths-based and gender-specific approach (Carroll et al., 2018, Robertson et al., 2013). The evaluation assessed effectiveness based on three research outcomes: (i) change in CVD risk, (ii) HBC measured using a Stages of Change (SoC) framework (Prochaska and Velicer, 1997) and, (iii) follow-up use of general practitioner (GP) services among those referred at baseline. Secondary outcomes were (i) to examine for ‘whom’ the HBC delivery methods worked, based on social, farming and health characteristics, and (ii) to provide evidence-based recommendations to support program implementation and scale-up. Although there have been previous cardiovascular health programs targeted at farmers internationally (Brumby et al., 2010, Jones and Fragar, 2008) and nationally (van Doorn et al., 2019, Evans et al., 2009, Evans et al., 2009, van Doorn et al., 2018) to our knowledge, no studies have developed such approaches for CVD prevention targeting specifically male farmers. Documenting these protocols is important to inform future gender-specific, community-based men’s health promotion programs targeting ‘at risk’ and ‘HTR’ groups.

## 2 Methods

### 2.1 Study approach

This study adopted a quasi-experimental effectiveness-implementation design (Curran et al., 2012). The FHH-CHP sought to evaluate and understand (i) the effectiveness of ‘real-world’ practice rather than the causal effect as a result of controlled conditions (Peters et al., 2013), and (ii) the generalizability of findings for this target population of farmers (Stuart et al., 2015) with the aim of program implementation and impacting policy and practice to improve farmers’ health. Whilst effectiveness studies have an internal validity limitation, these designs, specifically when conducted on multiple sites or based on a nationally representative sample, have greater external validity than a single-site RCT (Maciejewski, 2020). Higher external validity is important for generalizability to other population groups or contexts (Ahmad et al., 2010). Based on the Transtheoretical Model of Health Behavior Change (Prochaska and Velicer, 1997), the SoC was used to assess participants’ readiness for change and to track their progress through the different Stages. Ethical approval was granted from the ethics committee of the South East Technological University, Ireland, and the study was registered in the International Standard Randomized Controlled Trial Number Register (ISRCTN26792329).

### 2.2 Overview of FHH-CHP

**Fig. 2:**
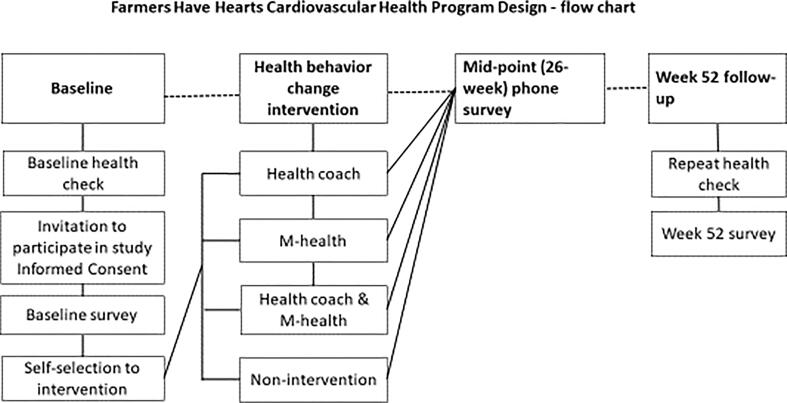
Farmers Have Heart Cardiovascular Health Program Flowchart.

The FHH-CHP consisted of a health check at baseline and Week 52, carried out by the NGO, and a HBC intervention, with a choice of three delivery methods (Fig. 2): health coaching by phone; mobile (M)-health by text messaging; health coaching and M-health combined. Farmers could also opt for the ‘usual care’ group, which comprised the health checks and data collection only. Data collection by surveys took place at baseline, Week 26 and Week 52. Two sub-groups of livestock farmers (‘cattle’ and ‘dairy’) were recruited from two different settings (‘livestock marts’ and’agri-branches’ respectively^1^). These two sub-groups represent different farming systems based on farm enterprise, size, and income (Donnellan et al., 2020).

**Fig. 3:**
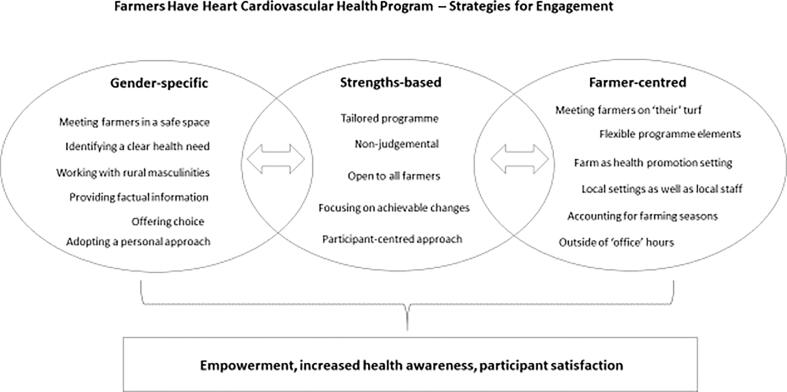
Farmers Have Heart Cardiovascular Health Program – Strategies for Engagement.

The design of the HBC intervention was informed by research evidence from men’s health (Carroll et al., 2018, Robertson et al., 2013) and farmers’ health (van Doorn et al., 2019, Brumby et al., 2009) and utilized strengths-based, non-clinical, informal and targeted approaches to respond to a defined health need (Fig. 3). This included ‘simple’ behavior change techniques, factual content and tailored resources (Robertson et al., 2013). All program elements were non-judgmental, i.e. accounting for individual health (behavior) choices and thus respected farmers’ autonomy and control over their health. Allowing for the unpredictability of farming, the delivery methods were flexible, practical and time convenient for participants (van Doorn et al., 2019). The lifestyle change domains included diet, physical activity, stress management and responsible drinking (Piepoli et al., 2016). Smokers who expressed a desire to stop smoking were referred to a free of charge national smoking cessation intervention for tailored and specialized support.

### 2.3 Sample size

Study participants were recruited from a cohort of 1400 farmers; 700 agri-branch and 700 mart farmers who attended free of charge cardiovascular health checks in the South and Midlands of Ireland, between May 2018-April 2019. Baseline health check recruitment at the marts was opportunistic and based on ‘self-referral’ by farmers attending the mart that day. Recruitment strategies included posters being displayed in advance at marts, and engagement efforts by staff of the NGO partner to personally invite/encourage farmers to participate. Recruitment for the baseline health checks at the agri-branches was coordinated by the agri-food business, which used different communication channels (an interactive online portal, text messaging and a specially commissioned information leaflet) to invite farmers to attend. These farmers pre-booked their preferred location, date and time.

The sample size calculation focused on recruiting a representative sample with CVD risk based on (i) a workforce of 33,370 male farmers in the selected regions (Central Statistics Office. Farm Structure Survey, 2016). A representative research sample size was 385 (expected population proportion of 0.5, confidence level 95 %–5 % error) (Conroy, 2015). Allowing for attrition, a minimum target study sample of n = 481 (1.25*384), was set.

### 2.4 Baseline and Week 52 health checks

Baseline and Week 52 health checks were conducted by nurses working for the NGO and included various clinical objective measurements (blood pressure (BP), lipid profile, blood glucose, Body Mass Index (BMI) and waist circumference). Blood pressure was measured using a calibrated Omron M or Omron 7 device in compliance with standardized measurement protocols. In cases of elevated BP readings, a repeat measurement was taken later on the other arm. Blood lipids and blood glucose were measured using a calibrated Alere Choltech LDX machine to analyze a finger prick blood sample. Heavy bulky clothing was removed for weight and waist measurements. Height was measured without shoes. BMI was calculated as weight in kilograms divided by the square of the height in meters (kg/m2) (World Health Organization, 2020). All measurements were recorded in a Results Booklet, a copy of which was provided to each farmer. On completion of all health check measurements, those farmers who warranted further medical attention, were given a standardized referral letter to give to their GP. Based on their baseline health check results, farmers received tailored lifestyle advice from the nurse using Motivational Interviewing techniques (Miller and Rollnick, 2002). All farmers received information about local health and support services and relevant health booklets, including a farmers’ health booklet (Osborne et al., 2016). A typical health check took 30 min.

Participants were invited for a repeat health check at Week 52 having indicated their preferred mode of notification (post, email or text message) at baseline. An invitation protocol was developed to maximize the response rates, which included a standardized methodology: all farmers booked their repeat health check two weeks in advance of their scheduled date. Mart farmers were invited to book through the research team and agri-branch farmers through the Agri-food business customer care service. Non-respondents were followed up by phone after five working days. A script was developed to counter anticipated reasons for declining the repeat health check (e.g. not having made changes to lifestyle; perceived lack of improvement in health; not having time). All farmers who booked in for a repeat health check received a reminder text the day before and a call on the morning of the health check. This resulted in a 61.7 % (n = 455) follow-up participation rate.

### 2.5 Health behavior change delivery methods

#### 2.5.1 Health coach

Health coaching facilitates HBC by supporting people to gain personal insight, knowledge and confidence (Peterson, 2006). With the target group of farmers in mind, a moderate number (six) of intervention phone sessions were chosen for reasons of practicality and time-efficiency (Hammersley et al., 2015). The sessions commenced with an introductory call approximately-four weeks after the baseline health check. All sessions were carried out by the same health coach, a health promotion and men’s health professional. Respecting farmers’ autonomy and choice, the health coach was not privy to the health check results and therefore could not ‘direct’ participants’ choice of lifestyle change domain(s). Behavior change strategies were based on Motivational Interviewing (Miller and Rollnick, 2002) and included techniques adapted from the ‘Behavior Change Wheel‘ (Michie et al., 2014) such as feedback on behavior, health consequences and goal setting (Table 1). An intervention handbook was developed with information on data protection, data collection, and a description of the SoC, including a scoring assessment and Stage relevant HBC techniques. To ensure intervention consistency, all sessions were guided by standardized questionnaires. Specifically developed administration forms were used to track the dates of phone calls, whether farmers had responded to the calls, and the intervention sequence to monitor completion of sessions.

**Table 1:** Overview of intervention elements, behavioral strategies and targeted construct*.

**Intervention element**	**Frequency**	**Description**	**Behavioral strategy**	**Gender sensitivity strategy**	**Targeted construct**
Cardiovascular risk factor screening	2x	A cardiovascular risk factor screening took place at baseline and Week 52. The FHH-CHP health checks were partly funded by the national health service, and carried out by a national health charity. The health screenings included measurement of cholesterol and blood glucose by point of care testing (Cholestech LDX), blood pressure, carbon monoxide, BMI and waist circumference measurements, as well as self-reported lifestyle risk factors such as family history, alcohol consumption (number of standard drinks per week), physical activity and stress.	Motivational interviewing Tailored lifestyle advice Raising cardiovascular health awareness Rolling with resistance Pros / cons of behavior for cardiovascular health	All nurses were trained in adopting a strengths-based and gender-sensitive approach to engaging farmers Positive, non-judgmental and strength-based content Use of appropriate language	Health literacy Knowledge Self-efficacy
Health booklets	2x	As part of the cardiovascular risk factor screening, participants were given relevant health booklets about specific risk factors for CVD such as: ‘Healthy Eating’, ‘Managing your Cholesterol’, ‘Managing your Stress’, ‘Managing your high Blood Pressure’, ‘Be Active’ and ‘Quit Smoking’. Additionally, all farmers received the health booklet ‘Staying fit for farming’ which was specifically targeted at farmers.	Raising cardiovascular health awareness Practical advice on health behavior change	Factual Solution based approach Use of appropriate language	Health literacy Knowledge Self-efficacy
Self-monitoring tools	2x	As part of the cardiovascular risk factor screenings, participants received self-monitoring tools such as a waist centimeter and a food diary.	Self-regulation of cardiovascular risk factors	Solution based approach	Self-efficacy
Health coach by phone	6 x	Participants were contacted by the health coach 6 times over a period of nine months. The health coach had previous experience with health promotion and had finalized the Engage National Men’s Health Training. The health coach intervention focused on support and encouragement of behavior change. Participants decided the domains of change with a main focus on diet, physical activity (PA), stress management and/or alcohol consumption. The health coach used motivational interviewing during the sessions.	Improved health awareness on diet, PA, stress management and/or alcohol consumption in relation to cardiovascular health. Rolling with resistance Provide feedback on behavior and health impacts Provide support and encouragement Encourage social support	Trained in applying a gender sensitive approach Participation led intervention Focus on autonomous decision making in relation to health behavior change Goal setting Positive, non-judgmental and strengths-based content Use of appropriate language	Self-efficacy Confidence Health literacy Knowledge Social support
M−health	34 text messages over 4 months	The M−Health text messaging intervention consisted of 34 messages per topic for the intervention: healthy eating, physical activity, stress management and responsible alcohol consumption. The text messages were personalized and the topics were tailored to the needs of the participants, which was discussed during an introduction call.	Improved knowledge Support and encouragement	Regular prompts for health behavior change Goal setting Positive, non-judgmental and strengths-based content Use of appropriate language	Self-efficacy Confidence Health literacy Knowledge

*Adapted from Carroll et al., 2019.

#### 2.5.2 M-health

The appeal of M-health by text messages lies in it being a fast and inexpensive health intervention with a wide reach (Abroms et al., 2015). The M-health group also commenced approximately-four weeks after the baseline health check with an introductory phone call. This outlined the choice of text message topics and also included validated measures of the SoC (Center for Substance Abuse Treatment, 1999). To keep costs low and to enhance future scale-up opportunities, an ‘off the shelf’ text messaging application was used. A text message library was developed based on health promotion materials (Richardson et al., 2015, Teagasc and Mental Health Ireland, 2017). The text messages used ‘everyday’ words, active verbs and short sentences (National Adult Literacy Agency, 2011), and each message included an ‘opt-out’ option. Farmers who opted-out stopped receiving text messages but continued the overall intervention and were invited for the Week 52 health check. Farmers received 3–4 text messages per week for four months (Fjeldsoe et al., 2014). The text messages were tailored based on first name and topic(s). The initial welcoming message also provided participants with contact details for the research team should they feel the need to talk in person, and SMART (specific, measurable, achievable, realistic, time-based) goals for consideration before they received the topic-specific text messages i.e. those that focused on the behaviors the farmer wanted to change.

#### 2.5.3 Health coaching in combination with M-health

Farmers could also opt for a combination of the health coach and M-health concurrently. The health coach conducted the introductory call and took note of the preferred topics for the text messages.

#### 2.5.4 Usual care group

Finally, farmers could opt for the ‘usual care’ group, which was confined to participation in the baseline and Week 52 health check as well as research data collection.

### 2.6 Study participation

Study participants were recruited from eligible health check attendees (male farmers, ≥18 years). The health checks were open to farmers, their families and mart workers. Female participants, non-farming participants, farmers with underlying illnesses impacting their mental capability (such as Alzheimer’s Disease) and farmers who were advised to seek immediate medical attention as a result of the health check, were excluded from study participation. Some 1319 baseline health checks were carried out during the study period and 1005 farmers met the inclusion criteria, of whom, 86.4 % (n = 868) consented to take part in the study.

Intervention allocation was based on self-selection. While self-selection raises the limitation of potential bias, choice on the other hand has been linked to increased feelings of autonomy, control and empowerment, values which are deemed particularly important by our target population (Hammersley et al., 2021) and suited a real world application and a pragmatic approach (Table 2).

**Table 2:** Real world application of Farmers Have Hearts Cardiovascular Health Program (FHH-CHP)*.

**Real world intervention characteristics**	**Specification**
Supported by existing practice and best evidence	The intervention is in line with best evidence of ‘what works with men’ and builds on learnings from the Farmers Have Hearts Evaluation 2013/14. Self-selection formed the basis for intervention which mimics real world situations in which people choose the treatment intervention most suitable to them and their lifestyle.
Underpinned by policy	The intervention addresses policy action point 2.8 from the Healthy Ireland Men’s Action Plan: ‘Implement the Farmers Have Hearts evaluation recommendations in the future roll-out of cardiovascular risk screening targeted at men’. It contributes to the implementation of Goal 6 of the National Farm Safety Partnership Action Plan (2016–2018) to promote improved health and wellbeing among the farming community.
Stakeholder collaboration	This intervention has been a unique collaboration between two academic institutes, Teagasc, Irish Heart Foundation, the National Health Service Executive and global Agri-food business Glanbia Ireland. This working partnership broadened the reach, acceptability and impact of the program as well as it enhances future opportunities for implementation and upscaling of the FHH-CHP.
Piloting of intervention	The Farmers Have Hearts Program Evaluation 2013/14 was used as a pilot for the current research in relation to engaging with farmers in a health screening program.
Cost effectiveness	To enhance implementation opportunities and in line with a pragmatic approach, the health interventions were simple in design and made use of easily available products and applications. The health coaching intervention emphasized support and encouragement rather than a medical and behavioral advice to ensure future implementation of the intervention by trained ‘lay’ people. The research was part of the evolution of an existing workplace health check program for farmers.
Simple	The intervention was straight forward and time effective. Farmers only had to use their phone to receive support in making lifestyle changes. They could choose their own time and pace to engage with the intervention. Engagement in the cardiovascular risk factor screening took place in a local workplace venue for farmers (agri-branch or livestock mart). Farmers felt at home, safe and respected in these venues, were met in a personal and friendly manner, which resulted in a lowering of potential barriers to taking part in the screening.
Self-selection	In line with ‘real-world’ situation: participants self-selected the intervention of their choice. This enhanced feelings of autonomy, greater adherence to interventions and more successful outcomes at a participant level.
Application opportunities to the ‘real world’	The intervention working protocols, cost effectiveness and ‘real world’ structures offer a range of possibilities for implementation of the intervention beyond the current phase of the study.

*Adapted from Carroll et al., 2019.

### 2.7 Data collection measures

#### 2.7.1 Baseline self-report measures

The baseline variables (Supplement 1) included socio-demographic and farming information, lifestyle factors, self-reported health, use of prescribed medication, use of GP services, overall experience of the health check, and cooking and dietary habits. Questions were based on previously validated national health and farming survey tools (Central Statistics Office. Farm Structure Survey, 2016, Health Service Executive: Healthy Ireland Survey Documents, 2018). Socio-demographic and farming characteristics included age (continuous), and categorical variables measuring marital status, living alone or with other(s), farming full time/part time, education level, farm size and farm enterprise. Lifestyle risk indicators included smoking (currently smoking; y/n); alcohol consumption (number of standard drinks consumed per week with an additional question on ‘harmful’ drinking) (O'Shea et al., 2017); physical activity (PA; occupational, leisure time y/n, with an open question on sedentary time); stress (a single validated question with five ordinal response categories); and well-being (the Short Warwick-Edinburgh Mental Well-being Scale with responses converted to a metric total score) (Warwick Medical School, 2018). Self-reported health included a Likert scale (1–10) measuring health importance and a categorical variable measuring health status. Current use of prescribed medication for BP, cholesterol and/or diabetes, medication adherence, whether respondents had a GP and time since last GP visit were measured categorically. Categorical questions explored reason(s) for taking part in the health check and whether respondents took part of their own volition or with prompting from others. A Likert scale (1–10) was used to measure respondents’ experiences of the health check, which was followed by an open question prompting them to explain their rating. Cooking and dietary habits were measured categorically (Yusuf et al., 2004). Farmers were also asked if they were contemplating HBC as a result of taking part in the health check (y/n).

#### 2.7.2 Week 26 phone follow-up questionnaire

A phone questionnaire (Supplement 2) at Week 26 focused primarily on HBC. A categorical question determined participants’ SoC and their lifestyle domain(s) of change (diet, PA, stress, alcohol consumption) whilst the Readiness for Change ruler (Center for Substance Abuse Treatment, 1999) measured participants’ motivation for HBC. Open questions recorded specific details of HBC. Categorical variables (van Doorn et al., 2017) were used to estimate follow-up use of GP services and new/altered medication for cholesterol, diabetes and/or BP. Open questions tracked outcomes of GP visits/barriers to accessing GP (van Doorn et al., 2017). Categorical questions (Wilder and Brumby, 2012) explored whether farmers had read, shared with other household members or could recall key messages from the health booklets (van Doorn et al., 2019). Further categorical questions explored whether farmers accessed family or community supports to make lifestyle changes whilst Likert-type scale (1–5) questions queried farmers’ perspectives on goalsetting, and whether they felt in control and able to take responsibility for lifestyle changes (Lee et al., 2008).

#### 2.7.3 Week 52 follow-up questionnaire

The Week 52 face-to-face survey (Supplement 3) further explored HBC and respondents’ experiences of the program. Categorical questions determined respondents’ description of their SoC; their motivation, sources of support and barriers to making lifestyle changes, domain(s) of change (diet, PA, stress, alcohol consumption); and their perceptions of how the intervention(s) impacted on HBC and personal health. The Readiness for Change ruler (Center for Substance Abuse Treatment, 1999) was used to measure motivation for and confidence in maintaining HBC. Overall experiences of the FHH-CHP were measured using a 5-point Likert scale. These focused on assessing motivating and support factors for health behavior change, evaluation of program elements, and overall experiences of taking part in FHH-CHP.

#### 2.7.4 Health coach and M-health questionnaire

The health coach and M-health interventions were preceded by an introductory questionnaire (Supplement 4) which included validated questions to assess respondents’ position relative to the SoC. These included the Readiness for Change Ruler (Center for Substance Abuse Treatment, 1999) and the domain(s) of HBC. The health coach sessions were based on respondents’ responses to a standardized questionnaire which accounted for the SoC (Center for Substance Abuse Treatment, 1999), goal setting (Michie et al., 2009) and self-efficacy (Lee et al., 2008), and tracked the domain(s) of change. During the M-health, respondents were asked five questions, based on the Readiness for Change Ruler, (Center for Substance Abuse Treatment, 1999) goal setting (Michie et al., 2009) and intervention rating.

### 2.8 Statistical analysis

Cardiovascular risk indicators were: BP ≥140/90 mmHg; total cholesterol ≥5.0 mmol/L; HDL cholesterol <1.0 mmol/L; LDL cholesterol ≥3.0 mmol/L; triglycerides ≥1.7 mmol/l; non-fasting blood glucose levels, ≥7.0 mmol/L, BMI kg/m^2^ ≥25.0–29.9, waist circumference ≥103 cm,^2^ current smoking (y/n); alcohol consumption ≥17 standard drinks per week (Health Service Executive, 2019), physical inactivity (<five days a week and <30 min on active days) (Department of Health and Health Service Executive, 2009) stress (often/very often) and consumption of <5 portions of fruit and/or vegetables a day (Yusuf et al., 2004).

Descriptive results were reported as frequencies, proportions, means and standard deviation or median and interquartile range for the whole group and subgroups. The primary study outcomes, change in CVD risk indicators and HBC based on the SoC between Week 26 and week 52 and the type of changes (diet, PA, stress, alcohol consumption) were assessed by comparing baseline to week 52, while the uptake of GP services was assessed at weeks 26 and 52. Differences in categorical variables, were assessed using Chi Square and McNemar tests, while change in continuous variables was assessed with t tests or appropriate non-parametric tests. Regression analysis (top-down models) was applied to understand program effectiveness for subpopulations of farmers, based on social and farming characteristics.

## 3 Discussion

The purpose of this protocol paper is to detail how the methodology used in the design, implementation and evaluation of a strengths-based and gender-specific health behavior change program (FHH-CHP) translated into ‘real world’ settings with a ‘HTR’ and ‘at risk’ population group (male livestock farmers): The intervention took place in farmers’ ‘workplace’, offered different options to ‘real’ intervention users, and allowed for daily unpredictable situations to happen in the evaluation. Establishing appropriate protocols is critical to ensuring that the translation of research into practice is replicable and can be disseminated at a population level. To our knowledge, this is the first study to adopt health coach, M-health and combined methods to target cardiovascular HBC among farmers. The findings of this study will therefore build upon a rich vein of gender-specific and strengths-based approaches to men’s health research (McGrath et al., 2021). Whilst carried out in Ireland, when taking into account the social, cultural and farming context, this study protocol can be used internationally. Findings will inform strategies on how to engage not just farmers, but potentially other ‘HTR’ groups, including but not limited to male-dominated industries, in public health interventions and to sustain their engagement over time.

The ‘real world’ conditions in which the FHH-CHP was ‘tested’ will provide robust evidence in (i) applying flexible strategies to meet the unpredictable nature of farming practices, (ii) establishing the effectiveness and replicability of the program with a view to potential national scale-up, and (iii) developing guidelines that inform gender-specific workplace health promotion interventions not just for farmers but for other occupational groups. A multi-actor partnership approach offered opportunities to create a wider community health support network to disseminate and implement learnings through different platforms. Additionally, the learnings can help frame a more holistic approach to farmer health and safety.

Respecting participants’ autonomy was in keeping with a ‘real world’ context, in which individuals actively chose a method that best suited their lifestyle and personal circumstances. When presented with the options, farmers carefully considered factors which might have impacted their commitment such as time requirements, mobile phone signal coverage, and the ‘usefulness’ to their personal situation. This approach also challenged more traditional rural masculinity norms, such as adhering to ‘good farmer’ values: being stoic, self-sufficient and a ‘provider’ (Hammersley et al., 2022); by reframing farmers active engagement in their own health as a socially acceptable, responsible and ‘manly’ choice, as well as normalizing health conversations within rural/farming settings (Hammersley et al., 2021). This approach was consistent with meeting farmers’ needs and maximizing the reach of the program to enable farmers to prioritize lifestyle changes to improve their cardiovascular health.

This study took a pragmatic approach (Porzsolt et al., 2015) in assessing program effectiveness in real world conditions. Thus allocation of participants to usual care or the enhanced HBC intervention arms was based on preference rather than randomization. If establishing causal association between the HBC intervention and final program outcomes is needed after program implementation, then conducting a RTC is recommended.

## 4 Conclusion

Research learnings can be used beyond health boundaries to influence other aspects of farming practice such as occupational health and safety, technology and practice adoption requiring farmer behavior change based on extension activities (advice provision and training). Findings will enhance current knowledge about the practicalities of targeted HBC programs aiming to reach and engage ‘at risk’ and ‘HTR’ groups’ as well as establishing the impact of such programs on these target groups. Such studies are vital in order to influence practice and to re-orientate services to meet critical needs, to build gender competency in service delivery and to address inequities in health outcomes experienced by specific sub-population groups of men.

## Funding

This study was made possible through financial support from Teagasc (Award number 2017082), South East Technological University Ireland, Irish Heart Foundation, Health Service Executive and Glanbia Ireland.

## CRediT authorship contribution statement

**Diana van Doorn:** Conceptualization, Methodology, Investigation, Data curation, Project administration, Formal analysis, Writing – original draft, Writing – review & editing. **Noel Richardson:** Conceptualization, Methodology, Writing – review & editing, Supervision. **David Meredith:** Conceptualization, Methodology, Writing – review & editing, Formal analysis, Supervision. **Catherine Blake:** Methodology, Investigation, Formal analysis, Supervision. **John McNamara:** Conceptualization, Methodology, Supervision.

## Declaration of Competing Interest

The authors declare that they have no known competing financial interests or personal relationships that could have appeared to influence the work reported in this paper.

## Data Availability

The data that has been used is confidential.

## Footnotes

1 An agri-branch is local farming supply retail outlet associated with an Agribusiness.

## References

[b0005] Abroms L.C., Whittaker R., Free C., Van Alstyne J.M., Schindler-Ruwisch J.M. (2015). Developing and pretesting a text messaging program for health behavior change: recommended steps. JMIR mHealth and uHealth.

[b0010] National Adult Literacy Agency, 2011. Simply put. Writing and Design Tips. Dublin. Report No.: 9781907171123.

[b0015] Ahmad N., Boutron I., Dechartres A., Durieux P., Ravaud P. (2010). Applicability and generalisability of the results of systematic reviews to public health practice and policy: a systematic review. Trials.

[b0020] Alberts B., Kirschner M.W., Tilghman S., Varmus H. (2014). Rescuing US biomedical research from its systemic flaws. Proc. Natl. Acad. Sci..

[b0025] Brumby S.A., Willder S.J., Martin J. (2009). The sustainable farm families project: changing attitudes to health. Rural Remote Health.

[b0030] Brumby S., Willder S., Martin J. (2010). Milking their health for all its worth?: improving the health of farming families through facilitated learning. Extens. Farming Syst. J..

[b0035] Carroll P., Harrison M., Richardson N. (2018). Evaluation of a gender-sensitive physical activity programme for inactive men in ireland: protocol paper for a pragmatic controlled trial. J. Phys. Activity Res..

[b0040] Center for Substance Abuse Treatment, 1999. TIP 35: Enhancing Motivation for Change in Substance Use Disorder Treatment. Rockville: Substance Abuse and Mental Health Services Administration (SAMHSA), U.S. Department of Health and Human Services. 22514841

[b0045] Central Statistics Office: Census of Agriculture, 2020. https://www.cso.ie/en/releasesandpublications/ep/p-coa/censusofagriculture2020-preliminaryresults/ Accessed 11/05/2022.

[b0050] Central Statistics Office. Farm Structure Survey, 2016. https://www.cso.ie/en/releasesandpublications/ep/p-fss/farmstructuresurvey2016. Accessed 09/05/2021.

[b0055] Conroy R. (2015).

[b0060] Curran G.M., Bauer M., Mittman B., Pyne J.M., Stetler C. (2012). Effectiveness-implementation hybrid designs. Med Care.

[b0065] Department of Health and Health Service Executive, 2009. The National Guidelines on Physical Activity for Ireland. Dublin:36–36.

[b0070] Donnellan T., Moran B., Lennon J., Dillon E. (2020). Teagasc national farm survey 2019 preliminary results. Teagasc.

[b0075] Dryden R., Williams B., McCowan C., Themessl-Huber M. (2012). What do we know about who does and does not attend general health checks? findings from a narrative scoping review. BMC Public Health.

[b0080] Eriksen, C.U., Rotar, O., Toft, U., Jørgensen, T., 2021 What is the effectiveness of systematic population-level screening programmes for reducing the burden of cardiovascular diseases? Copenhagen: World Health Organization Regional Office Europe. 33625816

[b0085] Evans, D., Walshe, K., Gillen, P., Connellan, M., 2009. Farmers Have Hearts Project Evaluation. Dublin: Department of Health, Health Promotion Services, Community Nutrition and Dietetics, Health Service Executive West.

[b0090] Evans D., Walshe K., Gillen P., Connellan M. (2009).

[b0095] Fjeldsoe B., Phongsavan P., Bauman A., Goode A., Maher G., Eakin E. (2014). ‘Get Healthy, Stay Healthy': protocol for evaluation of a lifestyle intervention delivered by text-message following the Get Healthy Information and Coaching Service®. BMC Public Health.

[b0100] Glasgow R.E., Emmons K.M. (2007). How can we increase translation of research into practice? types of evidence needed. Annu. Rev. Public Health.

[b0105] Hammersley M.L., Cann V.R., Parrish A.M., Jones R.A., Holloway D.J. (2015). Evaluation of the effects of a telephone-delivered health behaviour change program on weight and physical activity. Nutr. Diet..

[b0110] Hammersley C., Richardson N., Meredith D., Carroll P., McNamara J. (2021). “That’s me I am the farmer of the land”: exploring identities, masculinities, and health among male farmers’ in Ireland. Am. J. Men's Health.

[b0115] Hammersley C., Richardson N., Meredith D., Carroll P., McNamara J.G. (2022). Supporting farmer wellbeing: exploring a potential role for advisors. J. Agric. Educ. Extension.

[b0120] Health Service Executive, 2019. Alcohol Programme. https://www.hse.ie/eng/about/who/healthwellbeing/our-priority-programmes/alcohol-programme/ Accessed 14/09/2019.

[b0125] Health Service Executive: Healthy Ireland Survey Documents, 2018. https://www.gov.ie/en/collection/231c02-healthy-ireland-survey-wave/. Accessed 10/05/2018.

[b0130] Jones S., Fragar L. (2008). Community programs to improve cardiovascular health and cancer prevention A preliminary review of programs in rural Australia. Health (San Francisco).

[b0135] Lee S.Y., Hwang H., Hawkins R., Pingree S. (2008). Interplay of negative emotion and health self-efficacy on the use of health information and its outcomes. Commun. Res..

[b0140] Maciejewski M.L. (2020). Quasi-experimental design. Biostatistics Epidemiol..

[b0145] McGrath A., Murphy N., Richardson N. (2021). Study protocol: evaluation of sheds for life (SFL): a community-based men’s health initiative designed “for shedders by shedders” in Irish Men’s sheds using a hybrid effectiveness-implementation design. BMC Public Health.

[b0150] Michie S., Abraham C., Whittington C., McAteer J., Gupta S. (2009). Effective techniques in healthy eating and physical activity interventions: a meta-regression. Health Psychol..

[b0155] Michie S., Artkins L., West R. (2014).

[b0160] Miller W., Rollnick R. (2002).

[b0165] Osborne A., Carroll P., Richardson N., Doheny M., Brennan L., Lambe B. (2016). From training to practice: the impact of ENGAGE, Ireland's national men's health training programme. Health Promot. Int..

[b0170] O'Shea, J., Goff, P., Armstrong, R., 2017. SAOR Screening and Brief Intervention for Problem Alcohol and Substance Use.

[b0175] Peters D.H., Adam T., Alonge O., Agyepong I.A., Tran N. (2013). Implementation research: what it is and how to do it. BMJ.

[b0180] Peterson, D.B., 2006. People are complex and the world is messy: A behavior-based approach to executive coaching. Evidence Based Coaching Handbook: Putting best practices to work, 51–76.

[b0185] Piepoli M.F., Hoes A.W., Agewall S. (2016). 2016 European Guidelines on cardiovascular disease prevention in clinical practice: The Sixth Joint Task Force of the European Society of Cardiology and Other Societies on Cardiovascular Disease Prevention in Clinical Practice (constituted by representati. Eur. Heart J..

[b0190] Plueschke K., McGettigan P., Pacurariu A., Kurz X., Cave A. (2018). EU-funded initiatives for real world evidence: descriptive analysis of their characteristics and relevance for regulatory decision-making. BMJ Open.

[b0195] Porzsolt Franz F., Galito N., Toledo-Arruda A. (2015:). Efficacy and effectiveness trials have different goals, use different tools, and generate different messages. Pragmatic Observat. Res..

[b0200] Prochaska J.O., Velicer W.F. (1997). The transtheoretical model of health behavior change. J. Health Promot..

[b0205] Richardson N., Osborne A., O’Neill B. (2015). Staying fit for farming—a health booklet designed for Irish Farmers. J. Agromedicine.

[b0210] Robertson S., Witty K., Zwolinsky S., Day R. (2013). Men's health promotion interventions: what have we learned from previous programmes?. Commun. Practitioner.

[b0215] Warwick Medical School, 2018. The Warwick-Edinburgh Mental Wellbeing Scale (WEMWBS).

[b0220] Shaghaghi A., Bhopal R.S., Sheikh A. (2011). Approaches to recruiting 'hard-to-reach' populations into re-search: a review of the literature. Health Promot. Perspectives.

[b0225] Smyth B., Evans D.S., Kelly A., Cullen L., O'Donovan D. (2013). The farming population in Ireland: mortality trends during the 'Celtic Tiger' years. Eur. J. Public Health.

[b0230] Stuart E.A., Bradshaw C.P., Leaf P.J. (2015). Assessing the generalizability of randomized trial results to target populations. Prevent. Sci..

[b0235] Teagasc and Mental Health Ireland, 2017. Coping with The Pressures of Farming.

[b0240] van Doorn D., Richardson N., Osborne A. (2017). Farmers Have Hearts: the prevalence of risk factors for cardiovascular disease among a sub-group of Irish livestock farmers. J. Agromed..

[b0245] van Doorn D., Richardson N., Storey A. (2018). Farming characteristics and self-reported health outcomes of Irish farmers. Occup. Med..

[b0250] van Doorn D., Richardson N., Osborne A., Blake C. (2019). The impact of a workplace cardiovascular health screening programme 'Farmers Have Hearts' on health behaviour change among Irish farmers. Work.

[b0255] Vasileiou K., Barnett J., Thorpe S., Young T. (2018). Characterising and justifying sample size sufficiency in interview-based studies: systematic analysis of qualitative health research over a 15-year period. BMC Med. Res. Methodol..

[b0260] Wilder S., Brumby S. (2012). Health status and behaviours of Australian farming men. New Male Stud. Int. J..

[b0265] World Health Organization European Region Office, 2018. The health and well-being of men in the WHO European Region: better health through a gender approach.

[b0270] World Health Organization, 2020. http://www.euro.who.int/en/health-topics/disease-prevention/nutrition/a-healthy-lifestyle/body-mass-index-bmi Accessed 16/04/2020.

[b0275] Yusuf S., Hawken S., Ôunpuu S. (2004). Effect of potentially modifiable risk factors associated with myocardial infarction in 52 countries (the INTERHEART study): case-control study. Lancet.

