# Supplementary material for: Study protocol: Evaluation of the ‘real-world’ Farmers Have Hearts – Cardiovascular Health Program
Source: Prev Med Rep. 2022 Oct 17;30:102010. doi: 10.1016/j.pmedr.2022.102010 (PMC9747665; doi:10.1016/j.pmedr.2022.102010)
Supplement: Supplementary data 1 [file mmc1.rtf]

Farmers Have Hearts – Cardiovascular Health Programme - Baseline survey
Location ………………………….			Date ……………………………………. 
Thank you again for participating in the Farmers Have Hearts - Cardiovascular Health Programme. I would like to ask you a few questions to gather some background information for the study. This should not take longer than 5 minutes. All answers will be coded and anonymised. If there are any questions you do not want to answer, please let me know - that is no problem. Before we start, do you have any questions?
Intervention:   
Control group □1		M-health  □2 	Health Coach □3		Health coach and M-health □4
As a results of the health check, do you think of making change to your lifestyle to improve your heart health?
Yes  □1 		No  □2
If yes, what type of changes? 
Improve your Diet 		□1
Increase your Physical Activity	□2
Decrease your levels of Stress	□3
Reduce my alcohol intake		□4
Stop Smoking			□5 (Refer to national programme QUIT)
Other				□6 please specify ………………………..
A1	What age are you?       ……………..	
A2	What is your current marital status?
Married/co-habiting □1		Separated – divorced □3		In a relationship  □5
Single □2			 Widowed □4	
A3	Do you live alone? 
Yes  □1 		No  □2		


A4	What did your education include? 
Primary or below	  □1		Completed Leaving Cert  □3
Some secondary	□2		Third Level  □4	
A4a	Did you go to any formal Agricultural Education? 
Yes  □1 		No  □2	
A4b	If yes, What did your agricultural education include?
 Full-Time 3rd Level Agricultural Course  □1		Course > 60 hours  □5
Farm Apprenticeship Scheme  □2			Course < 60 hours  □6
Certificate in farming  □3				Other  □7 Please specify  …………..
1 year Agricultural College  □4	
A5	Is farming your full time or part time occupation?
Full time  □1		Part time  □2		
A5a	On average, how many hours do you farm per week? 	………………	
A5b


A5c	If part-time - on average, how many hours a week do you work OFF-FARM (e.g. contracting, working for another farmer, working for a building contractor). 		………………
Do you mainly farm on your own?
Yes  □1 		No  □2
Do you get help from family, friends or others running the farm
1.Never
2.Less than once a month
3.Roughly twice a month
4.Weekly
5.Daily	


A6	What do you consider to be your MAIN enterprise based on farm income (only one answer)?
Dairy  □1					Sheep / Drystock  □6
Dairy and cattle 	□2				Tillage  □7
Cattle rearing (suckler) 	□3			Tillage / drystock  □8
Cattle other (Drystock) 	 □4			Other  □9    ……………………
Mainly Sheep  □5	
A7	How many acres do you farm?
 0-25 Acres      □1			76 – 124 acres  □4
26 –50 acres   □2			125-248 acres   □5
51 – 75 acres  □3			≥249 acres        □6	
Self-reported health		
B1	On a scale from 1-10: how important is your health to you? (1 not - 10 very important)
1	2	3	4	5	6	7	8	9	10	
B2	How good is your health in general? 
Very Good   □1			Bad   □4
Good	      □2			Very Bad  □5
Fair               □3	
B3	Do you use any medication for cholesterol, blood pressure or diabetes?
Yes  □1 		No  □2	
B3a	For what condition do you use medication?  (multiple answers possible)
Cholesterol   □ 		Blood pressure	□ 	Diabetes    □	
B3b	Do you take the medication as prescribed by your doctor?
Yes  □1 		No  □2	
B3b.1 	If no, would you mind saying why not?
__________________________________________________________________	
B4	Do you have a GP?
Yes  □1 		No  □2	


B4a	When was the last time you visited your GP?
Within the last 4 weeks   □1			Over a year ago   □3
Within the last year (but over 4 weeks)   □2		I never visited my GP  □4	
B5	Do you have a medical card or GP visitor's card? 
Medical card    □1	GP visitors card   □2		No card   □3	
B5a	Do you have private health insurance?
Yes  □1 		No  □2	
	Health check 		
C1	Did you go on your own initiative to the heart health checks or were you encouraged by someone else?
Self   □1				Male friend   □4
Wife / partner   □	2		Female friend   □5
Children / family	□3		IH staff   □6	
C2 	Why did you decide to have the health check? (multiple answers possible)
Concerned about health  □1	 It was free  □4
Convenience   □2			Encouraged by others □5  
Curiosity  □3			Other   □6    ………….	

C3	On a scale of 1-10, how would you rate your experience of the health check (1 poor – 10 excellent)
1	2	3	4	5	6	7	8	9	10	
C3a	Would you mind explaining your rating? 
_________________________________________________________________________
_________________________________________________________________________	

	Lifestyle		
D1	Who does the majority of cooking in your household?
You   □1 				Family	□4
Wife/Partner  □2			Other	□5
Both my wife and I equally  □3	

D2	Choose what describes you best: 
A Do you eat snacks or salty foods one or more times a day?	
Yes  □1 		No  □2	
	B Do you eat deep fried foods or fast foods 3 or more times a week	Yes  □1 		No  □2	
	C Do you eat meat or poultry 2 or more times daily	Yes  □1 		No  □2	
	D Do you drink fizzy drinks one or more times a day?	Yes  □1 		No  □2	
	E Do you eat fruit one or more times daily	Yes  □1 		No  □2	
	If yes, on average, how many portions fruit per day?	……….	
	F Do you eat vegetables one or more times daily	Yes  □1 		No  □2	
	If yes, on average, how many portions vegetables per day?	……….	
