# Supplementary material for: Study protocol: Evaluation of the ‘real-world’ Farmers Have Hearts – Cardiovascular Health Program
Source: Prev Med Rep. 2022 Oct 17;30:102010. doi: 10.1016/j.pmedr.2022.102010 (PMC9747665; doi:10.1016/j.pmedr.2022.102010)
Supplement: Supplementary data 2 [file mmc2.docx]

**Farmers Have Hearts –Cardiovascular Health Programme - Week 26 questionnaire variables**

**Participant number:**

| **No** | **Variable** | **Questions** | **Answering Code** |
| --- | --- | --- | --- |
|  | **Section A** | **Health Behaviour change** |  |
| A1 | Changes made | As a result of your heart health check have you made any changes to your lifestyle such as your diet, physical activity, stress, alcohol consumption or smoking? | 1 Yes  2 No (go to A3b) |
| A1a | If yes | What type of changes have you made to your lifestyle? (Multiple answers possible) | 1 Improved my Diet  2 Increased my Physical Activity  3 Decreased my levels of Stress  4 Reduced the amount of alcohol that I drink  5 Stopped Smoking  6 Other: …..….. |
| A1b | If yes | Would you mind giving details on what changes you have made? | Open |
| A1c |  | Which of these lifestyle changes do you consider to be most important? (Please choose 1) | Open |
| A1d | If yes | Please answer what describes you best in relation to what stage you are at in terms of making changes to your lifestyle: | 1 I am thinking of making changes  2 I am in the process of making changes  3 I have made changes but still find it challenging  4 I have incorporated the changes fully in my daily life |
| A1c | If yes | What steps will you take to make these changes as part of your daily life long-term? | Open |
|  |  | **Stages of change** |  |
| A2 |  | On a scale from 1-10 how important is it for you to continue to make changes to your lifestyle [most important lifestyle change]? 1 = not and 10 very important | 1-10 |
| A2a |  | On a scale from 1-10 how confident are you that you can sustain this change in the longer term? | 1-10 |
| A3 | Health change | Have you noticed a change to your health as a result of making changes to your lifestyle? | 1 Yes  2 No |
| A3a | If yes | Can you describe what changes to your health you have noticed? | Open |
| A3b | If no | Are there any barriers that stopped you from making changes to your lifestyle? | Open |
|  | **Section B** | **Follow-up use GP services** |  |
| B1 | GP referral | As a result of the health check, were you advised to see your GP? | 1 Yes  2 No  3 Do not recall |
| B2 | Use of health services | Did you subsequently visit your GP? | 1 Yes  2 No (go to B3) |
| B2a | If yes | Would you mind sharing the outcome of your visit to the GP? | Open |
| B3 |  | As a result of the health check, did you visit any other health services? (prompt such as taking BP in pharmacy, counselling, consultant) | 1 Yes  2 No |
| B3a | Other health services | Please explain | Open |
| B4 | If no (but referred) | Can you identify any reasons or barriers that prevented you from visiting your GP? | Open |
| B4a | If no GP use but advised to see GP | Do you plan on attending your doctor/other health professional in the near future? | 1 Yes  2 No |
| B5 | Medication | Since the heart check, have you been prescribed medication for your heart, blood pressure or cholesterol or has your existing medication been altered? | 1 New medication  2 Medication has been altered  3 No |
| B5a | If yes | Do you take your medication as prescribed? | 1 Yes  2 No |
| **Note to researcher: if participant was referred to GP but cannot recall this or did not follow-up with their GP yet, advise him on the importance of visiting his GP.** | | | |
|  | **Section C** | **Health booklets and use of self-monitoring tools** |  |
| C1 | Health booklets | As part of the health check, did you bring home relevant health booklets? | 1 Yes  2 No  3 Don’t recall |
| C1a | If yes, | Did you read any of the health booklets which were made available to you? | 1 Yes  2 No |
| C1b | If yes | Was there a key message for you in a health booklet that made an impact on you? | 1 Yes  2 No |
| C1c | If yes | Can you specify your key message from the booklets? | Open |
| C2 |  | Did anyone in your family read any of the health booklets? | 1 Yes  2 No  3 Don’t know |
| C2a | If yes | Specify family member |  |
|  | **Section D** | **Social support** |  |
| D1 |  | Did you discuss making changes to your lifestyle to improve your heart health with others (such as your wife/partner, friends)? | 1 Yes  2 No |
| D1a | If yes, | Please specify with whom you discussed making changes to your lifestyle? | Open |
| D2 |  | Have you joined a class, e.g. keep fit, weightwatchers etc., club or other groups (e.g. men’s sheds) since you completed the health check? | 1 Yes  2 No |
|  | **Section E** | **Self-efficacy** |  |
|  |  | On a scale of 1-5, please answer what describes you best (1= not at all – 5= very much) |  |
| E1 |  | I am confident that I can have a positive effect on my lifestyle and health | 1-5 |
| E2 |  | I have set some definite goals to improve my health | 1-5 |
|  |  | I have been able to meet the goals I set for myself to improve my health | 1-5 |
|  |  | I am actively working to improve my health | 1-5 |
|  |  | I feel that I am in control of how and what I learn about my health | 1-5 |
|  | **Section F** | **Participation future research** |  |
|  | Future research | Do you give permission to be contacted by phone to share in further detail your experience of the FHH-CHP to take part in any future research on farmers' health? | 1 Yes  2 No |
| **Thank participant for their time and finish the phone call** | | | |
|  | | | |
