# Supplementary material for: Study protocol: Evaluation of the ‘real-world’ Farmers Have Hearts – Cardiovascular Health Program
Source: Prev Med Rep. 2022 Oct 17;30:102010. doi: 10.1016/j.pmedr.2022.102010 (PMC9747665; doi:10.1016/j.pmedr.2022.102010)
Supplement: Supplementary data 3 [file mmc3.docx]

**Supplement 3 FHH-CHP Week 52 questionnaire variables**

| **No** | **Variable** | **Questions** | **Code** |
| --- | --- | --- | --- |
|  |  |  |  |
| 2 |  | Participant number | Open |
| 3 |  | Location | Open |
| 4 |  | Date | m/d/yyyy |
| 5 | Intervention | Did you take part in any of the support interventions as part of the FHH-CHP programme, such as the health coach or text messages? | 1 Yes  2 No  3 Opted out  4 I intended to take part but didn’t start.  5 Other |
| 5a |  | If other, please specify | Open |
| 6 |  | If yes, what intervention? | 1 Text messages  2 Health coach  3 Text messages and health coach |
| A1 | Prevention | As a result of taking part in FHH-CHP, do you think you will go for a health check more regularly? | 1 Yes  2 No |
| A1a |  | If yes, how often do you think you will go for a health check? | 1 Yearly  2 Every 6 months  3 When not feeling well  4 Other |
| A1b |  | If other, please specify | Open |
| A1c |  | Are you likely to access any other type of health service in the future? | 1 Yes  2 No  3 Don’t know |
| A1d |  | If yes, please specify (Multiple answers possible) | 1 GP 2 Pharmacy  3 Health stands at ploughing championships  4 Consultant hospital  5 Other |
|  |  | If other, please specify | Open |
| B | **All participants** | **Stages of change** |  |
| B1 | Stage of change | As a result of FHH-CHP, have you made any changes to your lifestyle to improve your heart health? | 1 Yes I have made changes (Go to question B2)  2 I intended to make changes but didn’t succeed (go to question B5)  3 I made changes but did not sustain them (go to question B5)  4 No, and I did not want to make changes (Go to question B6) |
| B2 | Type of changes | If yes, what type of changes did you make? (Multiple answers possible) | 1 Improved my Diet  2 Increased my Physical Activity  3 Decreased my levels of Stress  4 Reduced the amount of alcohol that I drink  5 Stopped Smoking  6 Other |
| B2a |  | If other, please specify | Open |
| B3 | Changes to health | Do you notice a difference in your health as a result of making changes to your lifestyle? | 1 Yes  2 No  3 Don’t know |
|  | If yes | If yes, please specify | Open |
| B4 | Importance maintenance | If yes, on a scale of 1-10, how important is it for you to maintain these changes? | 1-10 |
| B4a | Confidence  Maintenance | If yes, on a scale of 1-10, how confident are you that you will maintain these changes? | 1-10 |
| B4b | Maintenance | If yes, what practical steps have you learned that have helped you to maintain these changes? (Go to section C) | Open |
| B5 | If answer 2 or 3 Contemplation / relapse | What stopped you from making/sustaining changes? (Go to question B7) | Open |
| B6 | If answer 4  Pre-contemplation | If no, would you mind explaining why not? | Open |
| B7 |  | As a result of taking part in FHH-CHP, are you thinking about making lifestyle changes to improve your heart health in the near future? (Go to section D) | 1 Yes  2 No  3 Maybe  4 Other |
|  |  | If other, please specify | Open |
| C | **If MADE changes** | **Motivating and supporting factors for making changes to lifestyle** |  |
|  |  | Please answer what describes you best. The following factors motivated and supported me to make lifestyle changes to improve my heart health |  |
| C1 |  | The support of family and friends | 1 Strongly agree  2 Agree  3 Neither agree / disagree  4 Disagree  5 Strongly disagree  6 N/A |
| C2 |  | The support from local community groups such as my sports club. Men’s shed, etc? | 1 Strongly agree  2 Agree  3 Neither agree / disagree  4 Disagree  5 Strongly disagree  6 N/A |
| C3 |  | Using self-monitoring tools such as a food diary, counting steps, waist centimetre etc. | 1 Strongly agree  2 Agree  3 Neither agree / disagree  4 Disagree  5 Strongly disagree  6 N/A |
| C4 |  | The support of the health coach intervention  *Researcher****: if participant reported having not taken part in the health coach intervention please fill in N/A.*** | 1 Strongly agree  2 Agree  3 Neither agree / disagree  4 Disagree  5 Strongly disagree  6 N/A |
| C5 |  | The support of the text messaging intervention  ***Researcher: if participant reported having not taken part in M-health intervention please fill in N/A.*** | 1 Strongly agree  2 Agree  3 Neither agree / disagree  4 Disagree  5 Strongly disagree  6 N/A |
| D | **For intervention participants. Those participants who reported not having taken part in health coach or M-health intervention to be excluded** | **Evaluation of what intervention elements contributed to health behaviour change**  In relation to the intervention as part of FHH-CHP; please answer what describes you best (if applicable, i.e. if participant took part in an intervention): |  |
| D1 | Frequency | The number of sessions and/or text messages were sufficient | 1 Strongly agree  2 Agree  3 Neither agree / disagree  4 Disagree  5 Strongly disagree  6 N/A |
| D2 | Understanding | It was easy to understand the content of the health coach and/or text messages | 1 Strongly agree  2 Agree  3 Neither agree / disagree  4 Disagree  5 Strongly disagree  6 N/A |
| D3 | Goal setting | The health coach and/or text messages helped me to set clear goals for lifestyle changes to improve my heart health | 1 Strongly agree  2 Agree  3 Neither agree / disagree  4 Disagree  5 Strongly disagree  6 N/A |
| D4 | Confidence | The health coach and/or text messages helped me with becoming more confident in making changes to my lifestyle changes to improve my heart health | 1 Strongly agree  2 Agree  3 Neither agree / disagree  4 Disagree  5 Strongly disagree  6 N/A |
| D5 | Positive | What did you like most about the intervention you took part in? | Open |
| D6 | Negative / improvement | What did you like least about the intervention you took part in? | Open |
| E | **All participants** | **Impact of taking part in the FHH-CHP** |  |
|  |  | Please answer what describes you best. As a result of taking part in the FHH-CHP, I now: |  |
| E1 | Increased knowledge | Know what I can do to prevent risk factors for heart disease? | 1 Strongly agree  2 Agree  3 Neither agree / disagree  4 Disagree  5 Strongly disagree |
| E2 | Prevention | Try to prevent health problems before I feel any symptoms? | 1 Strongly agree  2 Agree  3 Neither agree / disagree  4 Disagree  5 Strongly disagree |
| E3 | Responsibility | Feel more responsible for my own health | 1 Strongly agree  2 Agree  3 Neither agree / disagree  4 Disagree  5 Strongly disagree |
|  | All participants | On a scale of 1-5 how would you rate the different elements of the FHH-CHP |  |
| F | **All participants** | **Rating of different intervention elements** |  |
| F1 |  | Heart health checks | 1-5 |
| F2 | Score 6 when not applicable | Health coach | 1-6 |
| F3 | Score 6 when not applicable | Text messages | 1-6 |
| F4 |  | The overall experience of FHH-CHP from start to finish | 1-5 |
|  |  |  |  |
| G | **All participants** | **FHH-CHP** |  |
| G1 | Reason to participate | What was your main reason for taking part in the FHH-CHP? Multiple answers possible | 1 The prospect of receiving two free heart health checks  2 To get support with making lifestyle changes to improve my heart health  3 To help out with a research  4 To help other farmers in the future  5 Other, please specify |
| G2 | Recommendation | To what extent do you agree with the following statement: I would recommend FHH-CHP to my friends or family. | 1 Strongly agree  2 Agree  3 Neither agree / disagree  4 Disagree  5 Strongly disagree |
| H | Receiving results when available | Are you interested in receiving results from the study by post when they are available? | 1 Yes  2 No |
