# Supplementary material for: Study protocol: Evaluation of the ‘real-world’ Farmers Have Hearts – Cardiovascular Health Program
Source: Prev Med Rep. 2022 Oct 17;30:102010. doi: 10.1016/j.pmedr.2022.102010 (PMC9747665; doi:10.1016/j.pmedr.2022.102010)
Supplement: Supplementary data 4 [file mmc4.docx]

| **Steps** | | **FHH-CHP WORKSHEET HEALTH COACH - FIRST INTRODUCTION SESSION** |
| --- | --- | --- |
| **Participant code:** | | |
|  | | **Welcome and rapport**  Discuss health check with participant |
| **A**  **A1**  **A1a**  **A1b**  **A1c**  **A2**  **A2a** | | **Section A - Behaviour change**  *This section is to establish the most important lifestyle domain that the participant would like to change to improve his heart health. Additionally, we want to find out if there are other lifestyle domains he would like to make changes to. The first question will give insight into the stage of change (no = pre-contemplation).*  As a result of the health check, have you thought about making changes to your lifestyle?  Yes □1 No □2 (go to A1c)  If yes, what do you consider to be the most important change that you could make to improve your heart health?  Improve my Diet □1  Increase my Physical Activity □2  Decrease my levels of Stress □3  Reduce the amount of alcohol that I drink □4  Stop Smoking □5 (Refer to national programme QUIT)  Other □6 please specify ………………………..  What do you hope to achieve with this lifestyle change?  …………………………………………………………………………………………………………………….  If no, would you mind to explaining why you did not consider any changes (once the respondent has answered this question go to section B).  …………………………………………………………………………………………………………………….  Are you thinking of making other changes to your lifestyle at the same time?  Yes □1 No □2  If yes, which areas would you like to change:  Improve my Diet □1  Increase my Physical Activity □2  Decrease my levels of Stress □3  Reduce the amount of alcohol that I drink □4  Stop Smoking □5 (Refer to national programme QUIT)  Other □6 please specify ……………………….. |
| B  B1  B2  B3  B4 | | **Section B - General health self-efficacy questions**  *Before moving forward, ask you a few general questions for background information. Please ask the participant to answer what describes them best.*  :  My risk of getting heart disease is  Very low 🞏1 low 🞏2 high 🞏 very high 🞏  If I change [lifestyle domain] it would reduce my risk of heart disease  Not at all true 🞏1 hardly true 🞏2 somewhat true 🞏3 very true 🞏4  I am certain I can stick to change [lifestyle domain] even when I am busy (farming or otherwise)  Not at all true 🞏1 hardly true 🞏2 somewhat true 🞏3 very true 🞏4  On a scale of 10: how confident are you that you will be able to make change to [identified most important lifestyle change] over the next 3 months? (1=not at all confident – 5 = somewhat confident – 10 = extremely confident)  1 2 3 4 6 6 7 8 9 10 |
| C  C1  C2  C3  C4  C5 | | **Section C - Intentions for change**  *Discuss the identified most important lifestyle domain for change with the participant in more detail. Talk over the challenges and benefits of the lifestyle change as well as possible barriers, impact of others such as family and peers (positively and negatively) and applying the change in different settings (such as on farm, mart / co-op, parties, pub, local GAA club, etc). Afterwards ask the following question to get insight into the level of intention for change.*  On a scale from 1-7, how satisfying do you think changing your [identified most important lifestyle change of choice] for the next three months would be?  1 2 3 4 5 6 7  Not at all satisfying very satisfying  On a scale from 1-7, do you think that most people who are important to you will approve of your [identified most important *lifestyle change of choice*] for the next three months  1 2 3 4 5 6 7  Disagree Completely agree  On a scale from 1-7, do you agree that changing your *[*identified most important *lifestyle change of choice*] is up to you?  1 2 3 4 5 6 7  Disagree Completely agree  On a scale from 1-7, how determined are you to change [identified most important *lifestyle change of choice*] for the next three months  1 2 3 4 5 6 7  No intention High intention  On a scale from 1-7, how beneficial for your heart health of changing your [*lifestyle change of choice*] for the next three months  1 2 3 4 5 6 7  Not beneficial Highly beneficial |
| D  E1  E2  E3  E4  E4a  E5  E5a  E5b  E5c  E6 | **Section D – If participant opted for the health coach AND m-health intervention, please ask for the topics of the text messages the participant would like to receive. (Smoking is outside of the scope if this intervention and therefore not a topic).**  Diet □1  Physical Activity □2  Stress management □3  Alcohol consumption □4  **Section E - TO BE FILLED IN BY HEALTH COACH**  This section is to document the Stage of Change, the behaviour techniques (as specific possible) applied and self-monitoring tools (if applicable) advised.  Stage of change participant  Pre-contemplation / contemplation □1  Preparation □2  Action □3  Maintenance □4  Behaviour change technique(s):  …………….…………………………………………………………………………………………………………………………………………  ………………………………………………………………………………………………………………………………………………………  Goal set for next month?  Yes □1 No □2  Use of self-monitoring tools advised?  Yes □1 No □2  If yes, please provide which tool:  …………….…………………………………………………………………………………………………………………………………………  ………………………………………………………………………………………………………………………………………………………  Does the participant wants to be called next month?  Yes □1 No □2 (Go to E5b)  If yes, is an appointment made for the next session?  Yes, we agreed a time and date? □1  No, we agreed to try an ‘opportunistic’ session □2  If no, what is the reason?  Want to skip a month □1  Wants to opt out of intervention □2  If the participant wants to opt out of the intervention, please provide reason why:  …………….…………………………………………………………………………………………………………………………………………  ………………………………………………………………………………………………………………………………………………………  Call duration | |
|  | Close of session | |

| **Steps** | | **FHH-CHP M-HEALTH INTRODUCTION CALL** |
| --- | --- | --- |
| **Participant code:** | | |
|  | | **Welcome and rapport**  Remind participant of location health check and agreeing to participate in the M-health (text messages) intervention to improve heart health. |
| **A**  **For researcher**  **A1**  **A1a**  **A1b**  **A1c**  **A2**  **A2a** | | **Behaviour change**  *This section is to establish the most important lifestyle domain that the participant would like to change to improve his heart health. Additionally, we want to find out if there are other lifestyle domains he would like to make changes to.*  As a result of the health check, have you thought about making changes to your lifestyle?  Yes □1 No □2 (go to A1c)  If yes, what do you consider to be the most important change that you could make to improve your heart health?  Improve my Diet □1  Increase my Physical Activity □2  Decrease my levels of Stress □3  Reduce the amount of alcohol that I drink □4  Stop Smoking □5 (Refer to national programme QUIT)  Other □6 please specify ………………………..  What do you hope to achieve with this lifestyle change?  …………………………………………………………………………………………………………………….  If no, would you mind to explaining why you did not consider any changes (once the respondent has answered this question go to section B).  …………………………………………………………………………………………………………………….  Are you thinking of making other changes to your lifestyle at the same time?  Yes □1 No □2  If yes, which areas would you like to change:  Improve my Diet □1  Increase my Physical Activity □2  Decrease my levels of Stress □3  Reduce the amount of alcohol that I drink □4  Stop Smoking □5 (Refer to national programme QUIT)  Other □6 please specify ……………………….. |
| B  B1  B2  B3  B4 | | **General health self-efficacy questions**  *Before moving forward, ask you a few general questions for background information. Please ask the participant to answer what he thinks describes him best.*  My risk of getting heart disease is  Very low 🞏1 low 🞏2 high 🞏 very high 🞏  If I change [lifestyle domain] it would reduce my risk of heart disease  Not at all true 🞏1 hardly true 🞏2 somewhat true 🞏3 very true 🞏4  I am certain I can stick to change [lifestyle domain] even when I am busy (farming or otherwise)  Not at all true 🞏1 hardly true 🞏2 somewhat true 🞏3 very true 🞏4  On a scale of 10: how confident are you that you will be able to make change to [identified most important lifestyle change] over the next 3 months? (1=not at all confident – 5 = somewhat confident – 10 = extremely confident)  1 2 3 4 6 6 7 8 9 10 |
| C  C1  C2  C3  C4  C5 | | **Intentions for change**  *Ask the following question to get insight into the level of intention for change in relation to the most important domain for change (A1a).*  On a scale from 1-7, how satisfying do you think changing your [identified most important lifestyle change of choice] for the next three months would be?  1 2 3 4 5 6 7  Not at all satisfying very satisfying  On a scale from 1-7, do you think that most people who are important to you will approve of your [identified most important *lifestyle change of choice*] for the next three months  1 2 3 4 5 6 7  Disagree Completely agree  On a scale from 1-7, do you agree that changing your *[*identified most important *lifestyle change of choice*] is up to you?  1 2 3 4 5 6 7  Disagree Completely agree  On a scale from 1-7, how determined are you to change [identified most important *lifestyle change of choice*] for the next three months  1 2 3 4 5 6 7  No intention High intention  On a scale from 1-7, how beneficial for your heart health of changing your [*lifestyle change of choice*] for the next three months  1 2 3 4 5 6 7  Not beneficial Highly beneficial |
| D | **TO AGREE WITH PARTICIPANT**  Over the next 4 months you will receive text messages on the following topics (see questions A1a and A2a):  Improve my Diet □1  Increase my Physical Activity □2  Decrease my levels of Stress □3  Reduce the amount of alcohol that I drink □4  Stop Smoking □5 (Refer to national programme QUIT)  Other □6 please specify ……………………….. | |
|  | Close of session and explain that the participant will be asked twice a month asked to reply to a question by text which will be yes / no or a number.  Thank you for your participation. | |
